# Myxoma Virus Combination Therapy Enhances Lenalidomide and Bortezomib Treatments for Multiple Myeloma

**DOI:** 10.3390/pathogens13010072

**Published:** 2024-01-12

**Authors:** Alpay Yeşilaltay, Dilek Muz, Berna Erdal, Türker Bilgen, Bahadır Batar, Burhan Turgut, Birol Topçu, Bahar Yılmaz, Burcu Altındağ Avcı

**Affiliations:** 1 Department of Hematology, Faculty of Medicine, Başkent University Istanbul, Istanbul 34662, Türkiye; 2 Department of Virology, Faculty of Veterinary, Tekirdağ Namık Kemal University, Tekirdag 59030, Türkiye; dilekmuz@nku.edu.tr; 3 Department of Medical Microbiology, Faculty of Medicine, Tekirdağ Namık Kemal University, Tekirdag 59030, Türkiye; berdal@nku.edu.tr; 4 Department of Nutrition and Dietetics, Faculty of Health Sciences, Tekirdağ Namık Kemal University, Tekirdag 59030, Türkiye; tbilgen@nku.edu.tr; 5 Department of Medical Biology, Faculty of Medicine, Tekirdağ Namık Kemal University, Tekirdag 59030, Türkiye; bbatar@nku.edu.tr; 6 Department of Hematology, Faculty of Medicine, Tekirdağ Namık Kemal University, Tekirdag 59030, Türkiye; burhanturgut@hotmail.com (B.T.); dr.avciburcu@gmail.com (B.A.A.); 7 Department of Biostatistics, Faculty of Medicine, Tekirdağ Namık Kemal University, Tekirdag 59030, Türkiye; topcubirol@gmail.com; 8 Department of Tumor Biology and Immunology, Institute of Health Sciences, Tekirdağ Namık Kemal University, Tekirdag 59030, Türkiye; baharbora91@gmail.com

**Keywords:** multiple myeloma, oncolytic virus, myxoma virus, lenalidomide, bortezomib

## Abstract

This study aimed to explore the effectiveness and safety of *Myxoma virus* (MYXV) in MM cell lines and primary myeloma cells obtained from patients with multiple myeloma. Myeloma cells were isolated from MM patients and cultured. MYXV, lenalidomide, and bortezomib were used in MM cells. The cytotoxicity assay was investigated using WST-1. Apoptosis was assessed through flow cytometry with Annexin V/PI staining and caspase-9 concentrations using ELISA. To explore MYXV entry into MM cells, monoclonal antibodies were used. Moreover, to explore the mechanisms of MYXV entry into MM cells, we examined the level of GFP-labeled MYXV within the cells after blocking with monoclonal antibodies targeting BCMA, CD20, CD28, CD33, CD38, CD56, CD86, CD117, CD138, CD200, and CD307 in MM cells. The study demonstrated the effects of treating Myxoma virus with lenalidomide and bortezomib. The treatment resulted in reduced cell viability and increased caspase-9 expression. Only low-dose CD86 blockade showed a significant difference in MYXV entry into MM cells. The virus caused an increase in the rate of apoptosis in the cells, regardless of whether it was administered alone or in combination with drugs. The groups with the presence of the virus showed higher rates of early apoptosis. The Virus, Virus + Bortezomib, and Virus + Lenalidomide groups had significantly higher rates of early apoptosis (*p* < 0.001). However, the measurements of late apoptosis and necrosis showed variability. The addition of MYXV resulted in a statistically significant increase in early apoptosis in both newly diagnosed and refractory MM patients. Our results highlight that patient-based therapy should also be considered for the effective management of MM.

## 1. Introduction

Hematological malignancies, including lymphoma, leukemia, and multiple myeloma, have seen a recent rise in occurrence and mortality rates [1]. Multiple myeloma (MM), characterized as a malignancy originating from differentiated B cells, is the second most prevalent hematological cancer and constitutes approximately 1% of all newly diagnosed cancers [2]. The primary feature of MM is the excessive proliferation of malignant plasma cells (MPCs) in the bone marrow, which leads to clinical manifestations including skeletal complications, immunodeficiency, and anemia [3]. Chemotherapy forms the initial line of treatment for hematopoietic malignancies. However, the effectiveness of chemotherapy is limited due to drug resistance and the heterogeneous nature of these malignancies [4].

The insights gained from research on the underlying mechanisms of MM have brought about significant advancements in MM treatment approaches. Effective treatments have been developed in the last 20 years, including immunomodulatory drugs such as lenalidomide [5], proteasome inhibitors such as bortezomib [6], histone deacetylase inhibitors such as panobinostat [7], monoclonal antibodies such as daratumumab, isatuximab, and elotuzumab [8,9], and B-cell mature antigen-targeted chimeric antigen receptor T cells (CAR-Ts) [10]. These developments have brought about improvements in progression-free and overall survival rates. Despite this, the five-year survival rate of MM patients is about 52.3% [2]. In addition, treatment-free intervals are decreasing in patients with MM, and treatment-resistant disease remains common. Therefore, therapeutics with new mechanisms of action are needed to fully control the disease.

Oncolytic viruses (OVs) are natural or genetically modified viruses that do not infect normal cells but selectively infect malignant cells. Oncolytic virotherapy is a new approach that allows the use of OVs in tumor therapy. Although potential cancer suppression was identified after viral infections in the early 1900s, progress was rather slow due to concerns about the efficacy and safety of virotherapy [11]. However, with the development of genetic technology and virology in 1991, modified herpes simplex virus 1 (HSV-1) exhibited the ability to proliferate selectively in malignant cells and exhibited potent anti-tumor effects and gained increased interest in oncolytic virotherapy [12]. Since then, viruses such as adenovirus, reovirus, vaccinia virus, herpes simplex virus, measles virus, and Newcastle disease virus have been developed for oncolytic virotherapy [13,14,15]. Both DNA and RNA viruses have been used in oncolytic virotherapy [16,17]. The viruses used in oncolytic virotherapy are particularly promising for relapsed/resistant patients, as they eliminate malignant cells through mechanisms different from conventional chemotherapeutics [18,19].

Oncolytic viruses such as reovirus, measles virus, vaccinia virus, and vesicular stomatitis virus have been shown to have therapeutic potential for the treatment of MM [20,21,22,23,24,25]. *Myxoma virus* (MYXV) is classified in the *Leporipoxvirus* genus in the *Poxviridae* family, which has a double-stranded DNA genome. Having a strict tropism for rabbits and hares, MYXV causes no obvious pathology in either humans or mice [26,27]. The therapeutic effect of MYXV, an oncolytic virus whose therapeutic potential has recently been recognized, has been investigated in pancreatic cancer [28,29], melanoma [30,31], glioma [32,33], and rhabdoid tumor [34,35]. MYXV has also been shown to induce oncolysis by increasing apoptosis in myeloma cells [36]. Moreover, it has been observed that the intravenous injection of MYXV causes a reduction of 70–90% in tumor tissue [37].

MYXV does not rely on a specific cell surface receptor to bind to cells. Therefore, it can enter many different types of cells and initiate infection. However, MYXV cannot bind to and infect CD34+ hematopoietic stem cells [38,39]. The adhesion of Poxviruses such as MYXV to cells occurs in the form of virion binding mediated by proteins such as D8, A27, H3, and A26 encoded by the virus [40]. D8 binds chondroitin, A27 and H3 bind heparan, and A26 binds laminin [41,42,43,44]. Additionally, the binding of MYXV to cells involves its interaction with integrin 1 and CD98 receptor molecules and the subsequent further activation of several serine/threonine kinases [45,46]. Uncovering the pathways that mediate MYXV binding to and entry into cells is very important for oncolytic virotherapy [47].

A combination of several treatments has often been used to achieve the best results in current cancer treatment. Even OVs, which are effective therapeutics, still have limited efficacy when used alone. To increase the treatment efficacy of OVs, the use of OVs in combination with other treatment methods, such as chemotherapy, immunotherapy, and radiotherapy, is studied. This combination therapy will determine the treatment strategy for hematological malignancies in the future. Therefore, in this study, we primarily aimed to investigate the efficacy of MYXV alone and in combination with lenalidomide and bortezomib in MM cell lines and cells prepared from newly diagnosed and relapsed/refractory MM patients. In addition, we aimed to determine which apoptotic pathways were used by MYXV for oncolysis, as well as the mode of entry into myeloma cells.

## 2. Materials and Methods

### 2.1. Myxoma Virus and Cell Lines

MYXV was kindly gifted by the University of South Carolina School of Medicine, USA. MYXV is a recombinant virus with the expression of green fluorescent protein (GFP). BHK-21 (Baby Hamster Kidney-21) and Vero (African Green Monkey Kidney) cell lines were used for MYXV in vitro cultivation. Cells were incubated in an incubator with 5% CO_2_ at 37 °C using DMEM and GMEM media containing 10% fetal bovine serum (FBS), 1X penicillin–streptomycin, and 2 mM L-glutamine. The virus was purified and titrated as previously described [48].

U266 and MOPC-315 MM cell lines were provided by the American Type Culture Collection (ATCC, Manassas, VA, USA). MM cell lines were used as control cells in all virus infection assays. Enriched RPMI-1640 cell culture medium containing 15% FBS, 100 U/mL penicillin, 100 µg/mL streptomycin, 1% vitamins, 1% non-essential amino acids, and 5–10% sodium pyruvate was used for MOPC-315 and U266 cells. MM cell lines were incubated at 37 °C and 5% CO_2_. Lenalidomide and bortezomib were dissolved in dimethyl sulfoxide (DMSO) and stored at −20 °C as a stock solution (10 mg/mL, 38 mM) [49]. Bortezomib and lenalidomide were used at 13 nM and 10 µM concentrations, respectively, in MYXV + drug trial applications.

### 2.2. Bone Marrow Aspiration Samples

After obtaining informed consent, bone marrow aspiration samples were obtained from patients with MM who were diagnosed and followed up by Tekirdağ Namık Kemal University (TNKU) Health Practice and Research Hospital, Hematology Clinic. Since bone marrow samples taken from the patients were taken during routine examinations, no additional invasive procedures were applied to the patient. Approval was obtained for the study from the TNKU Medical Faculty Non-Interventional Ethics Committee (approval number 2018/116/08/07). For this purpose, a total of 30 MM patients who applied to the TNKU Faculty of Medicine Hematology outpatient clinic and were diagnosed with MM based on bone marrow aspiration and pathology results were included in the study. Of the patients, 16 were newly diagnosed symptomatic MM patients, and 14 were relapsed/resistant MM patients. Patients with active systemic infection, patients with advanced heart failure, patients who had undergone major surgery in the last 6 months, patients with bleeding diathesis, patients with secondary malignancies, patients with major psychiatric pathology, and patients who did not consent to participate in the study were not included in the study. A six-milliliter bone marrow aspiration sample was taken from the iliac crest of each patient under local anesthesia. The taken bone marrow aspiration material was subjected to the culture procedure, and all cultured cells were stocked at −80 °C.

### 2.3. Preparation of Primer MM Cells and Immortalization

Mononuclear cells were isolated from bone marrow samples using the Ficoll-histopaque gradient centrifugation method. Briefly, after adding 4 mL of Ficoll to the centrifuge tube, 6 mL of the bone marrow aspiration sample was layered and centrifuged at 400× *g* for 20 min. After centrifugation, the opaque interphase layer containing mononuclear cells was collected. Afterward, the interphase layer was transferred to a different centrifuge tube and washed in PBS. Trypan blue staining was used to determine the cell viability rate.

The Magnetic Cell Selector (MACS) method was performed for the positive selection of malignant plasma cells in bone marrow samples. CD138+ cells were separated using anti-CD138 antibodies and cultured. Briefly, CD138+ cells were marked with CD138 antibody MicroBeads and loaded onto the MACS column in a magnetic field. After keeping the labeled CD138+ cells in the column and removing the negative cells, the magnetic field was removed, and CD138+ cells were collected. The purity of the cells was checked by marking them with anti-CD138, anti-CD38, and anti-CD45 antibodies using flow cytometry. The isolated cells were cultured in enriched RPMI 1640 medium containing 15–20% FBS, 100 U/mL penicillin, 100 µg/mL streptomycin, 1% vitamins, 1% non-essential amino acids, and 5–10% sodium pyruvate incubated at 5% CO_2_ and 37 °C.

An immortalization protocol was applied to ensure the continuity of the weak-character cells in the subculture stages. The human telomerase reverse transcriptase (hTERT) method was used for the immortalization process of primary MM cells and was performed using the hTERT (pCI-neo-hEST2, Addgene, Watertown, USA) kit, as previously described [50].

### 2.4. RNA Isolation and cDNA Synthesis

After the transfection of primary cells, the cells were checked for the presence of sequences related to hTERT and plasmid by applying RNA extraction, cDNA synthesis, and PCR tests. After each passage, cells were frozen and thawed, and RNA extraction was performed. RNA isolation was carried out with the manufacturer’s protocol for the commercial RNA extraction kit (GeneJet RNA Purification Kit, Thermo, Waltham, MA, USA), and cDNA synthesis from the obtained RNA samples was studied by the manufacturer’s protocol for the cDNA Synthesis Kit (ReverAid First Strand cDNA Synthesis Kit, Thermo, Waltham, MA, USA).

### 2.5. Cell Viability Determination with WST-1

Water-Soluble Tetrazolium 1 (WST-1) was used to determine cell viability. In this study, eight cell groups per patient were formed to investigate the effects of MYXV and MYXV + drug combinations on cells. Group 1: cell control; Group 2: Bortezomib; Group 3: Lenalidomide; Group 4: Bortezomib + Lenalidomide; Group 5: MYXV; Group 6: MYXV + Bortezomib; Group 7: MYXV + Lenalidomide; and Group 8: MYXV + Bortezomib + Lenalidomide. All patient-derived cells were analyzed with the WST-1 assay for cell toxicity detection and flow analysis and ELISA tests for apoptosis analysis. U266 and MOPC-315 cells were used as control cells. Briefly, 100 µL of 4 × 10^4^ cells was transferred to each well in 96-well microplates. Then, PBS was added to the cell control. The cells were treated with 10 MOI MYXV, bortezomib at a concentration of 13 nM, and lenalidomide at a concentration of 10 µM. Cell lines were incubated for 24 and 48 h, followed by incubation with 10 µL of WST-1 solution for 3–4 h. The cells’ absorbance values were measured in a spectrophotometer at 420 nm, 450 nm, 480 nm, and 640 nm wavelengths. The ratio of the absorbance value to the control value was multiplied by 100 to obtain % cell viability.

### 2.6. Apoptosis Analysis

#### 2.6.1. Apoptosis Analysis by Flow Cytometry

Apoptosis analysis according to cell surface phosphoserine exposure was performed using flow cytometry. All cell groups for each patient were prepared using 1–2 × 10^6^ cells/mL and incubated for 48 h. The cells were stained with APC-Annexin V/PI (APC Annexin V Apoptosis Detection Kit with PI, BioLegend, San Diego, CA, USA) for 15 min at room temperature. After centrifugation, cell pellets were suspended in a buffer solution. All cells, apoptotic and necrotic, were analyzed with a flow cytometer (BD FACSCalibur, Franklin Lakes, NJ, USA).

#### 2.6.2. Caspase-9 Concentration Analysis by ELISA

The quantitative measurement of caspase-9 expression in MM cells was performed using the Human CASP9 (E-EL-H0663, Elabscience, Wuhan, China) ELISA kits, according to the manufacturer’s instructions. Briefly, standard dilutions and cell samples were added to wells and incubated at 37 °C for 90 min. Then, the liquid was removed from each well, and Biotinylated Detection Ab working solution (Elabscience) was added and incubated at 37 °C for 1 h. Then, the washing step was repeated 3 times, and HRP Conjugate working solution was added to each well and incubated at 37 °C for 30 min. After the washing step, the substrate was added to each well and incubated at 37 °C for 15 min. After stopping, the optical density (OD value) of the well was measured at 450 nm with a microplate reader (BioTek EL×800, Winooski, VT, USA). The caspase-9 concentration was calculated using the standard curve created according to the concentration and absorbance ratios.

#### 2.6.3. Identification of Surface Molecules That MYXV Uses for Entry into the MM Cell

The roles of 11 candidate cell surface molecules that MYXV could use for entry into MM cells were investigated. In this context, the functional roles of BCMA, CD20, CD28, CD33, CD38, CD56, CD86, CD117, CD138, CD200, and CD307 molecules were investigated to determine the cell surface molecules that play a role in the entry of MYXV into MM cells. The presence of MYXV was determined via flow cytometry using patient-derived primary MM cells or MM cell lines infected with 10 MOI of GFP-MYXV for 24 h. The GFP signal expressed intracellularly at the end of the incubation period was used to determine positive cells. According to the results obtained from preliminary experiments with different MOI amounts of the virus, an MOI of 10 MYXV was used. Purified MM cells from bone marrow samples from two different newly diagnosed MM patients and two different MM patients defined as refractory were inoculated into DMEM medium with 3 replications of 30,000 cells per well. After 24 h, monoclonal antibodies against the specific cell surface markers were added at 3 different concentrations based on the manufacturer’s recommendations. For the blocking experiments used antibody amount was named as “Middle”, “Low” (10 times below the medium concentration), and “High” (10 times above the medium concentration). The middle concentration values determined for the monoclonal antibodies used are given in Table 1. After treatment with monoclonal antibodies for 24 h, GFP-labeled MYXV at 10 MOI was added to each well and incubated for 48 h. At the end of the period, the collected cells were centrifuged with PBS at 1000 rpm for 3 min, and the supernatant was removed. The precipitated cells were suspended in 400 µL of PBS and analyzed using flow cytometry.

In flow cytometry, the negative control (cells not covered by any monoclonal antibodies and not infected with GFP-MYXV) and positive control (cells infected with GFP-MYXV and not blocked by monoclonal antibody positive control) were evaluated. Cell samples of the positive control and the tested antibodies were analyzed using flow cytometry, and the percentage of MYXV-positive cells was determined based on their GFP signals. During the analysis of the percentage values obtained, the percentage values for each of the investigated cell surface molecules were compared to the positive control values, and the change in the rate of infected cells was determined as the “percent change”. The effects of the investigated cell surface molecules on the intracellular entry of MYXV were then evaluated.

### 2.7. Statistical Analysis

The SPSS statistical package program (version 24) was used for statistical analysis. Descriptive statistics, such as the mean and standard deviation, and table and graph methods were used to present the results. Normality test analyses were performed to determine whether the variables were normally distributed. All statistical analyses were performed under the assumption that the variables did not show a normal distribution. The Mann–Whitney U test was used for the two-group comparisons of the variables. Fisher’s Exact Test was used to compare categorical variables. The Kruskal–Wallis test was used for three or more group comparisons of the variables. The Mann–Whitney U test with Bonferroni correction was used for subgroup comparisons. A *p*-value of less than 0.05 was considered statistically significant.

## 3. Results

Eight of the female patients had relapsed, and six were newly diagnosed; six of the male patients had relapsed, and ten were newly diagnosed patients. There was no statistically significant relationship between diagnosis (relapse/rediagnosis) and gender variables (*p* > 0.05). The mean age of relapsed patients was 66.42 ± 10.55 years, and it was not statistically different from the mean age of newly diagnosed patients of 61.36 ± 11.20 years (*p* = 0.248). Newly diagnosed patients were patients who had not received any treatment. Relapsed patients were patients who had received at least three lines of classical treatment, a proteasome inhibitor, two lines of IMID, and autologous stem cell transplantation.

### 3.1. Purification of MYXV

**Figure 1:**
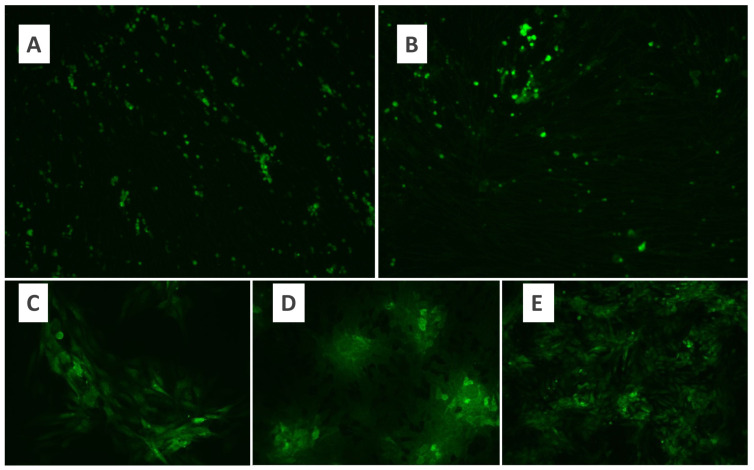
Fluorescence microscopy of growth of MYXV in the BHK-21 cell line (×10) (**A**), fluorescence microscopy of growth of MYXV in the Vero cell line (×10) (**B**), fluorescence microscopy of growth of MYXV in the Vero cell line. (**C**,**D**): ×20; (**E**): ×10.

**Figure 2:**
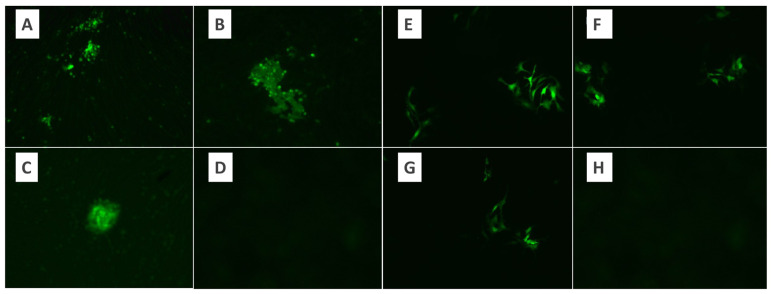
The appearance of virus growth foci under the fluorescence microscope in the microtitration test using the BHK-21 control cell line of MYXV. (**A**) Virus dilution ×10, (**B**,**C**) virus dilution ×20, (**D**) control. The appearance of virus growth foci under the fluorescence microscope in the microtitration test using the Vero control cell line of MYXV. (**E**–**G**) Virus dilution ×10. (**H**) Control.

BHK-21 and Vero cell lines were used for MYXV cultivation. Cell lines were evaluated under a fluorescence microscope, and MYXV growth was observed within the first 24 h. At the end of 48 h of the incubation period, fluorescent luminescence was observed in the majority of the cells (Figure 1). Therefore, a 48 h incubation period for MYXV was used throughout the study. In the microtitration test applied to determine the titer of MYXV, 10-fold logarithmic dilutions of MYXV were prepared, and quadruple repetitions were applied for each dilution. The tissue culture infective dose (DKID50) ratio was calculated by counting the growth foci of MYXV under a fluorescence microscope after incubation. The microtitration test was performed in duplicate for BHK-21 and Vero cells separately. As a result of MYXV growth, the MYXV titer obtained after the eighth passage of Vero cells was found to be higher than the titer obtained in BHK-21 cells (Figure 2). Both cells were defined as permissive for MYXV replication. Since MYXV grows in a shorter time in Vero cells, the MYXV growth foci count was performed after 48 h in the Vero cell line, while the MYXV growth foci count was performed after 72 h in the BHK-21 cell line. MYXV purification was performed in an ultracentrifuge (Beckman Coulter Optima XPN-100 and Beckman Coulter Max-XP) with 40%, 36%, 32%, 28%, and 24% sucrose solutions. The MYXV pellet obtained after centrifugation with gradual sucrose-gradient steps was diluted according to the protocol, and the OD values were recorded. After each purification protocol, the control of the presence and infectivity of MYXV from the MYXV stock, a titration test to calculate the titer, and dose calculations for further trials were performed. MYXV was diluted to 10 MOI and used in infection trials.

The infection rates with 10 MOI MYXV in U266 and MOPC human MM cell lines were 26.61% and 5.43%, respectively. In a total of 14 patients, with 7 newly diagnosed with MM and 7 with refractory MM, the rates of 10 MOI MYXV infecting MM cells were 40.25% (SD; 19.39) and 32.51% (SD; 8.98), respectively. There was no difference between the patient groups (*p* = 0.357).

### 3.2. WST-1 Results

From the results of WST-1 tests, the dose range in which MYXV affects the viability of cells most significantly was determined to be between 5 MOI and 10 MOI doses. The values at the 48th and 72nd hours of MYXV infection indicate decreased cell viability. Therefore, for primary cell lines, incubation with 10 MOI MYXV for 48 h was performed. Cell toxicity values in the applications with MYXV were observed to be lower than those in the control and other drug combination groups (Table 2). It was determined that the cell toxicity rates of the drugs in the Bortezomib, Lenalidomide, and Bortezomib + Lenalidomide administration groups were higher than those in the control, and the cell viability was lower than that in the control (*p* < 0.001).

### 3.3. MYXV Induces Cell Death via Apoptosis

**Figure 3:**
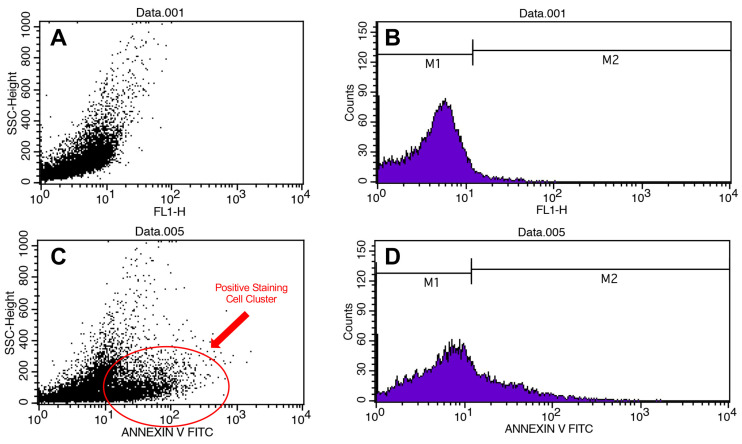
Flow cytometry results. Scatter plots (**A**) and histograms of cell counts (**B**) for negative control. Scatter plots (**C**) and histograms of cell counts (**D**) for positive control.

**Figure 4:**
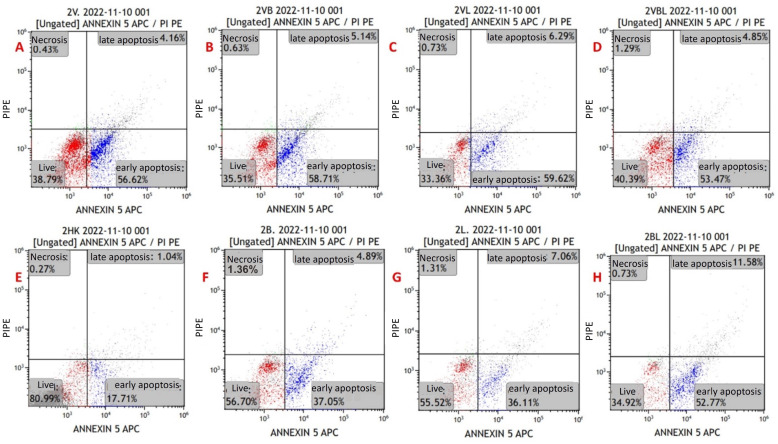
Annexin V/PI-staining flow cytometry image of apoptosis in myeloma cells from a newly diagnosed patient with MM. (**A**) MYXV-infected cell, (**B**) MYXV-infected cell combined with bortezomib, (**C**) MYXV-infected cell combined with lenalidomide, (**D**) MYXV-infected cell combined with bortezomib and lenalidomide, (**E**) control, (**F**) bortezomib-administered cell, (**G**) lenalidomide-administered cell, (**H**) bortezomib- and lenalidomide-administered cell. Live cells were visualized in the red part and apoptotic cells were blue part.

**Figure 5:**
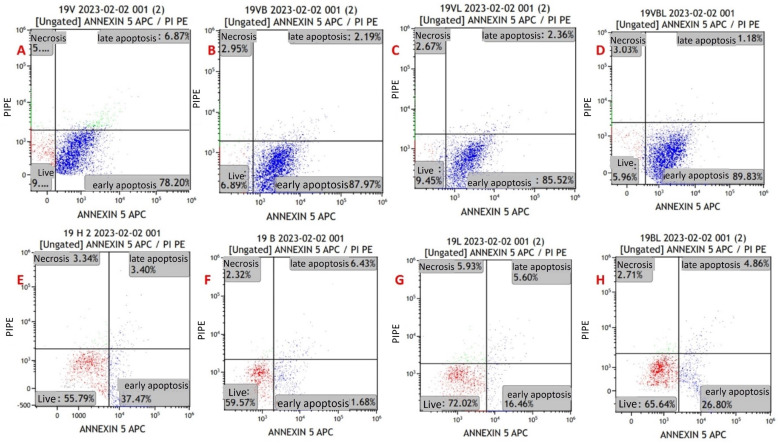
Annexin V/PI-staining flow cytometry image of apoptosis in myeloma cells from a refractory patient with MM. (**A**) MYXV-infected cell, (**B**) MYXV-infected cell combined with bortezomib, (**C**) MYXV-infected cell combined with lenalidomide, (**D**) MYXV-infected cell combined with bortezomib and lenalidomide, (**E**) control, (**F**) bortezomib-administered cell, (**G**) lenalidomide-administered cell, (**H**) bortezomib- and lenalidomide-administered cell. Live cells were visualized in the red part and apoptotic cells were blue part.

Apoptosis in the cell lines was evaluated via flow cytometry using the Annexin V/PI kit (Figure 3). Primarily, it was performed on MYXV-infected U266 and MOPC315 cell lines for optimization purposes. For the viability measurements, the results of all study groups were compared with the control group. The mean viability rates in the control were higher than the mean in the other groups. The decrease in viability in groups with MYXV presence was greater than in groups without MYXV (*p* < 0.001) (Figure 4 and Figure 5) (Table 3). The lowest cell viability was in the MYXV + Lenalidomide group, followed by the MYXV + Bortezomib, MYXV, and MYXV + Bortezomib + Lenalidomide groups, in that order. However, the survival rates in the Lenalidomide, Bortezomib, and Bortezomib + Lenalidomide groups in which MYXV was not added were similar to that in the control group. Similarly, the increase in the rate of apoptosis in the groups with the presence of MYXV was also higher than in the groups without MYXV.

Early apoptosis rates were found to be higher in the groups that were treated with MYXV than in the groups without MYXV (*p* < 0.001). The highest rates of early apoptosis were in the MYXV + Bortezomib + Lenalidomide group, followed by the MYXV, MYXV + Bortezomib, and MYXV + Lenalidomide groups, in that order (Table 3). Early apoptosis rates in the Bortezomib, Lenalidomide, and Bortezomib + Lenalidomide groups without MYXV were similar to that of the control group. There was no significant difference between the groups in terms of late apoptosis and necrosis rates (*p* > 0.05). In virus infection experiments in cell lines (U266 and MOPC-315), time-dependent increases in late apoptosis and necrotic cell density were detected at the 12th, 24th, 48th, and 72nd hours.

Viability and apoptosis assessments were also performed for newly diagnosed MM patients and refractory MM patients. In newly diagnosed MM patients, cell viability rates were found to be lower in the presence of MYXV than in the control (*p* < 0.001) (Table 4). The lowest cell viability rates were in the MYXV + Bortezomib group, followed by the MYXV + Bortezomib + Lenalidomide, MYXV + Lenalidomide, and MYXV groups, in that order (Table 4). Apoptosis rates were found to be higher in newly diagnosed MM patients in the presence of MYXV than in the control (*p* < 0.001). The highest rates of early apoptosis were in the MYXV + Lenalidomide group, followed by MYXV + Bortezomib, MYXV, and MYXV + Bortezomib + Lenalidomide groups, in that order (Table 4). There was no difference between the groups in terms of late apoptosis and necrosis rates in newly diagnosed MM patients. In patients with refractory MM, cell viability rates were lower in groups with MYXV than in groups without MYXV (*p* < 0.001) (Table 5). The lowest cell viability in patients with refractory MM was in the MYXV + Bortezomib + Lenalidomide group, followed by the MYXV + Bortezomib, MYXV, and MYXV + Lenalidomide groups, in that order. In patients with refractory MM, the rates of early apoptosis in MYXV groups were lower than those without MYXV (*p* < 0.001). The highest rate of early apoptosis in patients with refractory MM was in the MYXV + Bortezomib + Lenalidomide group, followed by the MYXV, MYXV + Bortezomib, and MYXV + Lenalidomide groups, in that order. There was no difference between the groups in terms of late apoptosis and necrosis rates in patients with refractory MM.

### 3.4. Analysis of Caspase-9 Expression

Caspase-9 levels in the MYXV + Bortezomib group were found to be borderline significantly higher in newly diagnosed MM patients than in patients with refractory MM (*p* = 0.081) (Table 6). However, there was no significant difference in caspase-9 levels between patients with newly diagnosed MM and patients with refractory MM in other groups. It was observed that the caspase-9 protein expression levels in the MYXV groups were significantly higher than the caspase-9 expression in the groups without MYXV in both newly diagnosed MM patients and refractory MM patients (*p* < 0.05) (Table 7).

### 3.5. MYXV Entry Pathways

Patient-derived samples were cultured with CD138+ selection. Flow analysis was used to check molecular expression during cell proliferation. Experiments were conducted with cells that had high expression after culturing. Monoclonal antibody blockade was performed for 11 different cell surface molecules using purified primary MM cells from two patients with refractory MM and two newly diagnosed MM patients. The rate of MYXV infection of MM cells was then compared with the positive control. The results are given in Table 8. It was determined that in patients with newly diagnosed MM, following monoclonal antibody blockade, the entry of MYXV into MM cells generally decreased compared to the control. In patients with refractory MM, following monoclonal antibody blockade, the entry of MYXV into MM cells generally increased compared to the control. With CD86 blockade, the entry of MYXV into MM cells was decreased in one patient with refractory MM and two newly diagnosed MM patients compared to the control. In the first patient with newly diagnosed MM, it was observed that MYXV entry into MM cells decreased compared to the control following CD28, CD38, CD117, CD138, and CD307 blockade. In the first patient with refractory MM, the entry of MYXV into MM cells increased compared to the control following CD200, BCMA, CD117, CD138, and CD307 blockade. In the second patient with refractory MM, the entry of MYXV into MM cells increased compared to the control following CD20, CD200, BCMA, CD28, CD38, CD117, and CD138 blockade.

When the data of newly diagnosed MM patients were combined and evaluated, there was no significant difference in MYXV entry into MM cells after monoclonal antibody blockade (*p* > 0.05). When the data of patients with refractory MM were combined and evaluated, a significant difference was determined for the entry of MYXV into MM cells after monoclonal antibody blockade only after low-dose CD86 blockade (*p* = 0.017, T-test). When the data from a total of four patients with newly diagnosed MM and refractory MM were combined and evaluated, there was no significant difference in MYXV entry into MM cells after monoclonal antibody blockade (*p* > 0.05).

The results indicate heterogeneity resulting from individual differences, and different cell surface molecules may play a role in the entry of MYXV into the MM cell in different patients. While multiple cell surface molecules may mediate the entry of MYXV into the cell, other cell surface molecules not included in this study may have a more important and specific role.

## 4. Discussion

In our investigation, we examined the effects of combining lenalidomide and bortezomib, two commonly employed treatments for MM worldwide, with MYXV. Our findings revealed a reduction in cell viability, an increase in early apoptosis, and an upregulation of caspase-9 expression in the groups treated with MYXV. However, we also observed considerable variability in the ability of MYXV to enter MM cells.

Because of the plasticity that leads to the emergence of resistant clones and tumor heterogeneity in the treatment of MM, a complete treatment cannot be performed. Despite the recent introduction of new treatment strategies, MM remains an incurable malignancy. Therefore, there is an active need for new therapeutic modalities in the treatment of MM. Recently, there have been suggestions in both experimental and clinical studies that OVs could be a potential therapeutic alternative to treat hematological malignancies [51,52,53]. OVs can be used for therapeutic purposes alone and/or in combination with standard chemotherapeutic agents [54].

Recently, the effectiveness of OVs on MM has been studied extensively. In a study with adenovirus, it was shown that myeloma cells are susceptible to CD40L-mediated apoptosis, and adenovirus treatment reduced the tumor burden by 50% in a xenograft mouse model [38]. In another study, it was determined that adenovirus serotype 5 was able to infect and kill most myeloma cell lines and ex vivo patient MPCs [55]. In studies with another OV, HSV-1 has been reported to infect myeloma cell lines and CD138+ primary cells, reduce the tumor volume after intratumoral injection [56], and exhibit enhanced antimyeloma effects in combination with lenalidomide [57]. Bartee et al. [36] determined that HSV1716 increased cell death by 50–80% in four human myeloma cell lines through the induction of FASL and proapoptotic genes such as caspase-1, -8, and -9. It was also observed that HSV1716 reduced the tumor burden by 50% in myeloma xenografts. Reovirus has been shown to increase cell death through both apoptosis and autophagy in myeloma cell lines and ex vivo tumor samples [58] while reducing the tumor burden and bone disease in xenograft models of myeloma without any adverse effects [11].

MYXV is a double-stranded DNA virus with oncolytic potential against many hematological malignancies, including MM [59]. Zhang et al. [60] showed that MYXV increases apoptosis in the human neuroglioma cell lines A172 and U251 in a dose- and time-dependent manner. A MYXV lacking the antiapoptotic protein M011L has been reported to increase apoptosis in murine brain-tumor-initiating cells and prolong survival in immunocompetent tumor-bearing mice in vivo [61]. Madlambayan et al. [62] showed that MYXV inhibited myeloid sarcoma development and bone marrow grafting of two human acute myeloid leukemia (AML) cell lines. Similarly, MYXV has been reported to target leukemia cells in AML tumor xenografts without harming normal hematopoietic stem cells [63].

Graft-versus-host disease that develops in allo-hematopoietic cell transplantations in MM is one of the most important obstacles to treatment. The infection of activated T cells with MYXV reduced their proliferation and production of proinflammatory cytokines, reducing graft-versus-host disease. Ex vivo virotherapy with MYXV appears promising for allo-hematopoietic cell transplantation [64]. In another study, it was documented that the intravenous administration of MYXV to mice with disseminated myeloma eliminated 70–90% of malignant cells within 24 h and that MYXV also induced CD8+ T-cell responses with potent antimyeloma effects [65]. In a recent study, autologous murine bone marrow carrier leukocytes pre-infected with MYXV were found to be therapeutically superior to free virus or MYXV-infected peripheral blood mononuclear cells [66]. In a study examining the effects of MYXV on MM cells, it was reported that the murine bortezomib-resistant Vk12598 cell line was completely susceptible to MYXV, and oncolytic MYXV alone or in combination with chemotherapy/immunotherapy was found to be effective for treating drug-resistant MM in vivo [53]. In our study, it was observed that 26.6% of U266 cells were infected and 5.43% of MOPC cells were infected following 48 h of incubation with 10 MOI MYXV. While there are no studies on the rate of MYXV infection of cells with MOPC, reports have shown that this rate varied between 45% and 75% in MM cell lines, such as HuNS1, MM.1S, and RPMI-8266 [26]. Reovirus has been reported to be highly sensitive to the myeloma cell lines RMPI-8226 and U226 but shows low sensitivity to the H929, L-363, and MM.1S cell lines [67]. The difference between the infection rates found in previous studies and the infection rates found in our study may be affected by many factors, ranging from the fact that each cell-line type has its own unique cell surface molecule profile to differences in the medium used and the negativity threshold detected in the flow cytometry analysis.

Focusing on preclinical studies, it seems that MYXV results are derived from MM cell lines rather than primary myeloma cells derived from patients with MM. In our study, we studied two MM cell lines and primary myeloma cells obtained from patients with newly diagnosed MM and refractory MM. We also evaluated the effect of MYXV on myeloma cells, both alone and in combination with drugs used in the treatment of MM, specifically bortezomib and lenalidomide. In applications with MYXV, cell toxicity values were found to be lower than in the control and other drug combination groups. In addition, cell viability was lower in the MYXV-treated groups than in the groups not treated with MYXV. Moreover, the rates of early apoptosis in the MYXV-treated groups were higher than in the groups not treated with MYXV. This effect of MYXV on cell viability and apoptosis was similar in both newly diagnosed MM and refractory MM patients. Since oncolytic viruses are organisms by nature, they do not have direct cytopathic effects on the cell upon encountering the cell. After entering the cell, the virus uses the cell to continue its proliferation within the cell, a finding that is expected to increase cell viability. Then, after reaching a certain concentration within the cell, it drives the cell into apoptosis. The WST-1 test is used to determine cellular cytotoxicity by measuring the reduction of WST-1 formazan compounds, which are associated with mitochondrial changes. The test showed that virus-related cytotoxicity is lower than that of chemotherapeutic drugs. This is because the virus allows the cell to survive for a while to replicate itself in the targeted cell, and then it undergoes apoptosis, causing the cell to die. In contrast, drugs can cause cell death directly through their toxic effects. These results confirm previous study results showing that MYXV increases cell death in MM cells. In addition, no differences were observed between the drug treatments bortezomib and lenalidomide given in combination with MYXV.

Dunlap et al. [68] determined that MYXV infection causes the inhibition of activating transcription factor 4, which is the primary mediator of apoptosis in MM cells. In a study in which human U266 MM cells were infected with 10 MOI MYXV, it was determined that apoptosis was triggered by the induction of caspase-8 initially and then caspase-9 [36]. In our study, we found that MYXV treatment increased caspase-9 expression in myeloma cells of both newly diagnosed and recurrent MM patients. Previous studies have shown that MYXV generally increases caspase-8. The increase in caspase-9 by MYXV obtained in our study is very important for further studies. These results should be validated by further molecular studies.

The interactions of myeloma cells with stromal cells and the extracellular matrix are very important for their survival [69,70]. CD28 expression in myeloma cells causes a worse prognosis [71]. The binding of the myeloma cell receptor CD28 to CD80/CD86 of an antigen-presenting cell (i.e., the CD80/CD86-CD28 interaction) provides an antiapoptotic signal via the phosphatidylinositol 3-kinase/Akt pathway [72]. By blocking this pathway, an increase in the killing of myeloma cells has been determined. Most myeloma cells express CD86 as well as CD28. In the study of Gavile et al. [73], it was revealed that CD86 is necessary for myeloma cell survival and drug resistance. Moreover, the cytoplasmic region of CD86 is important for triggering molecular changes, such as the upregulation of Interferon regulatory factor 4 and Integrin beta-1 in myeloma cells [74]. In our study, with the blockade of CD28, the entry of MYXV into myeloma cells was decreased in two of the four patients and increased in one. With CD86 blockade, three of the four patients had reduced entry of MYXV into myeloma cells. Our study results, when evaluated together with previous study results, suggest that CD86 may be an alternative for the treatment of myeloma cells with MYXV. Studies have shown that CD200 expression is increased in MM [75]. For this reason, CD200 MM has been considered as a new treatment parameter in recent years [76]. In our study, we observed that the rate of MYXV increased in myeloma cells from patients with refractory MM after CD200 blockade. In the future, the use of MYXV with the anti-CD200 antibody may be an alternative in the treatment of refractory MM.

There is still limited success in the treatment of MM. In particular, no monotherapy causes complete remission in MM, and in refractory/recurrent MM, a single chemotherapy drug often leads to drug resistance or even ineffectiveness. Recently, oncolytic virotherapy alone or in combination with other therapeutic strategies has shown promise. However, the interaction between different treatment strategies is complex. Sometimes, drug combinations antagonize each other, reducing therapeutic activity and even causing side effects [77]. For this reason, both the selection of the appropriate virus and its combination with the right drug are very important for treatment. Despite these limitations, OVs still hold promise for the treatment of hematological cancers.

In conclusion, we determined that MYXV increased cell death in both MM cell lines and primary myeloma cells obtained from patients with newly diagnosed MM and refractory MM. Cell death occurred especially in the early apoptosis period and with the increase in the caspase-9 expression level. We also determined that different cell surface molecules may play a role in the entry of MYXV into MM cells in different patients. This indicates heterogeneity in MM due to individual differences. Our study results show that patient-based therapy should also be considered for the effective treatment of MM.

## Figures and Tables

**Table 1:** Monoclonal antibodies and “Middle” concentration values used in the study.

Biolegend Catalog No.	Antibody	Determined “Middle” Concentration
302902	Purified anti-human CD28	0.5 µg/100 µL
302302	Purified anti-human CD20	2 µg/100 µL
356602	Purified anti-human CD38	0.5 µg/100 µL
323404	Purified anti-human CD117	0.5 µg/100 µL
303402	Purified anti-human CD33	0.5 µg/100 µL
362502	Purified anti-human CD56	4 µg/100 µL
329202	Purified anti-human CD200	2 µg/100 µL
374202	Purified anti-human CD86	0.5 µg/100 µL
357502	Purified anti-human CD269	4 µg/100 µL
356502	Purified anti-human CD138	0.5 µg/100 µL
340202	Purified anti-human CD307	0.5 µg/100 µL

**Table 2:** WST-1 test results in MM patient subgroups.

MM Patient	Groups	Mean ± S.D.	*p*-Value
Newly Diagnosed	MYXV	137.29 ± 40.45	0.001
	MYXV + Bortezomib	141.37 ± 49.17	0.001
	MYXV + Lenalidomide	139.81 ± 43.53	0.001
	MYXV + Bortezomib + Lenalidomide	152.56 ± 60.79	0.001
	Bortezomib	93.28 ± 8.85	0.001
	Lenalidomide	100.45 ± 11.33	0.735
	Bortezomib + Lenalidomide	96.03 ± 12.54	0.197
Refractory	MYXV	125.78 ± 17.81	0.000
	MYXV + Bortezomib	126.76 ± 22.74	0.001
	MYXV + Lenalidomide	129.81 ± 43.53	0.001
	MYXV + Bortezomib + Lenalidomide	126.57 ± 23.32	0.001
	Bortezomib	91.49 ± 8.28	0.001
	Lenalidomide	94.04 ± 8.24	0.006
	Bortezomib + Lenalidomide	90.43 ± 10.46	0.000

**Table 3:** Flow cytometry analysis results in all patients’ myeloma cells.

Groups	Viability		Early Apoptosis		Late Apoptosis		Necrosis	
	$\bar{X}\pm ss$	*p*-Value	$\bar{X}\pm ss$	*p*-Value	$\bar{X}\pm ss$	*p*-Value	$\bar{X}\pm ss$	*p*-Value
Control	69.23 ± 19.11	<0.001	17.13 ± 13.12	<0.001	5.85 ± 7.23	*p* > 0.05	7.76 ± 11.04	*p* > 0.05
Bortezomib	65.00 ± 23.51		24.82 ± 18.86		6.02 ± 6.74		4.16 ± 4.51	
Lenalidomide	69.54 ± 21.65		19.97 ± 16.46		5.23 ± 5.74		5.24 ± 5.35	
Bortezomib + Lenalidomide	66.18 ± 27.34		21.58 ± 19.93		6 ± 6.46		6.22 ± 13.21	
MYXV	41.76 ± 29.47 ^1b. 5c. 9b. 13b^		45.89 ± 27.09 ^1a. 5b. 9a. 13b^		5.98 ± 6.99		6.26 ± 10.06	
MYXV + Bortezomib	40.51 ± 27.55 ^2b. 6b. 10a. 14b^		45.42 ± 26.07 ^2a. 6b. 10a. 14b^		6.94 ± 8.16		7.12 ± 11.97	
MYXV + Lenalidomide	38.71 ± 26.85 ^3b. 7c. 11b. 15c^		44.32 ± 25.85 ^3a. 7c. 11b. 15b^		4.98 ± 5.46		7.74 ± 14.44	
MYXV + Bortezomib + Lenalidomide	54.23 ± 28.54 ^4a. 8b. 12a. 16b^		47.12 ± 25.08 ^4a. 8b. 12a. 16a^		6.78 ± 8.39		7.41 ± 11.03	

^1^ Control-MYXV, ^2^ Control-MYXV + Bortezomib, ^3^ Control-MYXV + Lenalidomide, ^4^ Control-MYXV + Bortezomib + Lenalidomide, ^5^ Bortezomib-MYXV, ^6^ Bortezomib-MYXV + Bortezomib, ^7^ Bortezomib-MYXV + Lenalidomide, ^8^ Bortezomib-MYXV + Bortezomib + Lenalidomide, ^9^ Lenalidomide-MYXV, ^10^ Lenalidomide-MYXV + Bortezomib, ^11^ Lenalidomide-MYXV + Lenalidomide, ^12^ Lenalidomide-MYXV + Bortezomib + Lenalidomide, ^13^ Bortezomib + Lenalidomide- MYXV, ^14^ Bortezomib + Lenalidomide-MYXV + Bortezomib, ^15^ Bortezomib + Lenalidomide-MYXV + Lenalidomide, ^16^ Bortezomib + Lenalidomide-MYXV + Bortezomib + Lenalidomide, ^a^ α < 0.001, ^b^ α < 0.01, ^c^ α < 0.05.

**Table 4:** Flow cytometry analysis results in newly diagnosed patients’ myeloma cells.

Groups	Viability		Early Apoptosis		Late Apoptosis		Necrosis	
	$\bar{X}\pm ss$	*p*-Value	$\bar{X}\pm ss$	*p*-Value	$\bar{X}\pm ss$	*p*-Value	$\bar{X}\pm ss$	*p*-Value
Control	78.00 ± 17.88	<0.001	17.43 ± 14.05	<0.001	2.24 ± 4.35	*p* > 0.05	2.31 ± 2.22	*p* > 0.05
Bortezomib	65.73 ± 19.49		18.75 ± 17.86		2.51 ± 4.18		3.05 ± 4.84	
Lenalidomide	78.21 ± 19.27		16.57 ± 16.45		2.47 ± 4.16		2.73 ± 3.22	
Bortezomib + Lenalidomide	78.94 ± 20.45		16.13 ± 16.9		2.58 ± 4.32		2.33 ± 2.78	
MYXV	52.67 ± 27.22 ^1c. 9c. 13c^		41.18 ± 24.19 ^1c. 5c. 9c. 13b^		3.03 ± 5.2		3.2 ± 4.91	
MYXV + Bortezomib	50.48 ± 24.71 ^2c. 6c. 10c. 14c^		42.42 ± 21.89 ^2b. 6c. 10b. 14b^		3.32 ± 6.02		3.77 ± 6.56	
MYXV + Lenalidomide	51.24 ± 23.29 ^3c. 7c. 11c. 15c^		43.04 ± 20.59 ^3b. 7c. 11b. 15b^		2.13 ± 3.88		3.57 ± 6.09	
MYXV + Bortezomib + Lenalidomide	50.8 ± 24.61 ^4c. 8c. 12c. 16c^		40.96 ± 20.08 ^4c. 8c. 12c. 16b^		4.06 ± 8.34		4.2 ± 6.8	

^1^ Control-MYXV, ^2^ Control-MYXV + Bortezomib, ^3^ Control-MYXV + Lenalidomide, ^4^ Control-MYXV + Bortezomib + Lenalidomide, ^5^ Bortezomib-MYXV, ^6^ Bortezomib-MYXV + Bortezomib, ^7^ Bortezomib-MYXV + Lenalidomide, ^8^ Bortezomib-MYXV + Bortezomib + Lenalidomide, ^9^ Lenalidomide-MYXV, ^10^ Lenalidomide-MYXV + Bortezomib, ^11^ Lenalidomide-MYXV + Lenalidomide, ^12^ Lenalidomide-MYXV + Bortezomib + Lenalidomide, ^13^ Bortezomib + Lenalidomide-MYXV, ^14^ Bortezomib + Lenalidomide-MYXV + Bortezomib, ^15^ Bortezomib + Lenalidomide-MYXV + Lenalidomide, ^16^ Bortezomib + Lenalidomide-MYXV + Bortezomib + Lenalidomide, ^b^ α < 0.01, ^c^ α < 0.05.

**Table 5:** Flow cytometry analysis results in refractory patients’ myeloma cells.

Groups	Viability		Early Apoptosis		Late Apoptosis		Necrosis	
	$\bar{X}\pm ss$	*p*-Value	$\bar{X}\pm ss$	*p*-Value	$\bar{X}\pm ss$	*p*-Value	$\bar{X}\pm ss$	*p*-Value
Control	59.21 ± 15.6	<0.001	16.79 ± 12.49	<0.001	9.97 ± 7.79	*p* > 0.05	13.99 ± 13.71	*p* > 0.05
Bortezomib	52.74 ± 22.15		31.77 ± 18.1		10.04 ± 6.98		5.43 ± 3.89	
Lenalidomide	59.63 ± 20.48		23.86 ± 16.18		8.37 ± 5.79		8.12 ± 5.93	
Bortezomib + Lenalidomide	51.60 ± 27.49		27.81 ± 21.86		9.9 ± 6.38		10.67 ± 18.46	
MYXV	29.28 ± 27.73 ^1c. 5c^		51.27 ± 30.06 ^1b^		9.35 ± 7.4		9.86 ± 13.11	
MYXV + Bortezomib	29.11 ± 29.94 ^2c. 6c^		48.85 ± 30.65 ^2c^		11.08 ± 8.5		10.95 ± 15.51	
MYXV + Lenalidomide	33.45 ± 28.3 ^8c^		45.78 ± 31.58 ^3c^		8.25 ± 5.27		12.51 ± 19.4	
MYXV + Bortezomib + Lenalidomide	24.90 ± 27.07 ^4b. 7b^		54.16 ± 28.95 ^4b. 7c^		9.88 ± 7.58		11.05 ± 13.83	

^1^ Control-MYXV, ^2^ Control-MYXV + Bortezomib, ^3^ Control-MYXV + Lenalidomide, ^4^ Control-MYXV + Bortezomib + Lenalidomide, ^5^ Lenalidomide-MYXV, ^6^ Lenalidomide-MYXV + Bortezomib, ^7^ Lenalidomide-MYXV + Bortezomib + Lenalidomide, ^8^ Bortezomib + Lenalidomide-MYXV + Lenalidomide, ^b^ α < 0.01, ^c^ α < 0.05.

**Table 6:** Comparison of caspase-9 levels in groups with MYXV and those without MYXV in MM patients.

	Multiple Myeloma	Mean ± S.D.	*p*
Control	Newly Diagnosed	2.90 ± 0.55	0.299
	Refractory	2.34 ± 0.29	
Bortezomib	Newly Diagnosed	2.76 ± 0.28	0.803
	Refractory	2.79 ± 0.41	
Lenalidomide	Newly Diagnosed	2.32 ± 0.26	0.852
	Refractory	2.44 ± 0.36	
Bortezomib + Lenalidomide	Newly Diagnosed	2.30 ± 0.27	0.349
	Refractory	2.16 ± 0.39	
MYXV	Newly Diagnosed	5.42 ± 0.62	0.135
	Refractory	5.86 ± 1.81	
MYXV + Bortezomib	Newly Diagnosed	6.55 ± 1.00	0.081
	Refractory	5.95 ± 2.06	
MYXV + Lenalidomide	Newly Diagnosed	5.84 ± 0.86	0.383
	Refractory	6.73 ± 2.41	
MYXV + Bortezomib + Lenalidomide	Newly Diagnosed	6.95 ± 0.85	0.158
	Refractory	7.85 ± 2.99	

**Table 7:** Comparison of caspase-9 in groups without MYXV in both newly diagnosed MM patients and refractory MM patients

	Newly Diagnosed MM		Refractory MM	
	Mean ± S.D.	*p*	Mean ± S.D.	*p*
Control	2.90 ± 0.55	0.001	2.34 ± 0.29	0.001
MYXV	5.27 ± 0.62		5.86 ± 1.81	
Bortezomib	2.76 ± 0.28	<0.001	2.79 ± 0.41	0.013
MYXV+ Bortezomib	6.56 ± 1.00		5.95 ± 2.06	
Lenalidomide	2.32 ± 0.26	<0.001	2.44 ± 0.36	0.001
MYXV + Lenalidomide	5.64 ± 0.86		6.73 ± 2.41	
Bortezomib + Lenalidomide	2.30 ± 0.27	<0.001	2.16 ± 0.39	<0.001
MYXV + Bortezomib + Lenalidomide	6.91 ± 0.85		7.85 ± 2.99	

**Table 8:** Changes in infection rates in refractory MM and newly diagnosed MM patients after monoclonal antibody blockade. In the table, those with a statistically significant increase in the MYXV ratio compared to the control are shown in green, and those with a statistically significant decrease are shown in red.

	Refractory Patient 1	Refractory Patient 2	Newly Diagnosed Patient 2	Newly Diagnosed Patient 2
CD33_Low	0.120	0.619	0.029	0.000
CD33_Middle	0.202	0.058	0.084	0.016
CD33_High	0.011	0.352	0.020	0.009
CD86_Low	0.001	0.134	0.012	0.014
CD86_Middle	0.002	0.153	0.046	0.004
CD86_High	0.002	0.217	0.040	0.005
CD20_Low	0.004	0.551	0.083	0.296
CD20_Middle	0.001	0.002	0.313	0.086
CD20_High	0.037	0.030	0.052	0.095
CD200_Low	0.001	0.001	0.079	0.091
CD200_Middle	0.003	0.016	0.185	0.150
CD200_High	0.017	0.006	0.242	0.270
CD56_Low	0.063	0.154	0.158	0.454
CD56_Middle	0.002	0.004	0.011	0.222
CD56_High	0.028	0.064	0.003	0.054
BCMA_Low	0.233	0.390	0.091	0.169
BCMA_Middle	0.003	0.016	0.028	0.333
BCMA_High	0.002	0.007	0.048	0.546
CD28_Low	0.349	0.002	0.046	0.236
CD28_Middle	0.014	0.008	0.038	0.066
CD28_High	0.009	0.026	0.576	0.017
CD38_Low	0.668	0.056	0.011	0.344
CD38_Middle	0.004	0.023	0.014	0.033
CD38_High	0.119	0.022	0.003	0.725
CD117_Low	0.032	0.277	0.011	0.042
CD117_Middle	0.022	0.015	0.014	0.135
CD117_High	0.045	0.013	0.020	0.070
CD138_Low	0.022	0.468	0.006	0.200
CD138_Middle	0.004	0.005	0.009	0.210
CD138_High	0.004	0.021	0.004	0.373
CD307_Low	0.014	0.022	0.009	0.078
CD307_Middle	0.002	0.530	0.003	0.075
CD307_High	0.009	0.083	0.018	0.076

## Data Availability

The data that support the findings of this study are available from the corresponding author upon reasonable request.
